# Preparation and Research on Mechanical Properties of Eco-Friendly Geopolymer Grouting Cementitious Materials Based on Industrial Solid Wastes

**DOI:** 10.3390/ma17153874

**Published:** 2024-08-05

**Authors:** Zhonglin Li, Ye Xu, Chengzhi Wu, Weiguang Zhang, Yang Chen, Yibing Li

**Affiliations:** 1 Department of Materials Science and Engineering, Guilin University of Technology, Guilin 541004, China; dahe121133@163.com (Z.L.); xuye121306@163.com (Y.X.); wuchengzhi73@gmail.com (C.W.); zhangwg@glut.edu.cn (W.Z.); chengyang2313@glut.edu.cn (Y.C.); 2 Collaborative Innovation Center for Exploration of Nonferrous Metal Deposits and Efficient Utilization of Resources, Guilin University of Technology, Guilin 541004, China; 3 Key Laboratory of New Processing Technology for Nonferrous Metals and Materials, Ministry of Education, Guilin University of Technology, Guilin 541004, China

**Keywords:** red mud, GGBS, geopolymer, grouting materials

## Abstract

Red mud (RM), a hazardous solid waste generated in the alumina production process, of which the mineral composition is mainly hematite, is unable to be applied directly in the cement industry due to its high alkalinity. With the rise of geopolymers, RM-based grouting materials play an essential role in disaster prevention and underground engineering. To adequately reduce the land-based stockpiling of solid wastes, ultrafine calcium oxide, red mud, and slag were utilized as the main raw materials to prepare geopolymers, the C-R-S (calcium oxide–red mud–slag) grouting cementitious materials. The direct impact of red mud addition on the setting time, fluidity, water secretion, mechanical properties, and rheological properties of C-R-S were also investigated. In addition, a scanning electron microscope (SEM), X-ray diffraction (XRD), three-dimensional CT (3D-CT), Fourier transform infrared spectroscopy (FT-IR), and other characterization techniques were used to analyze the microstructure and polymerization mechanism. The related results reveal that the increase in red mud addition leads to an enhanced setting time, and the C-R-S-40 grouting cementitious material (40% red mud addition) exhibits the best fluidity of 27.5 cm, the lowest water secretion rate of 5.7%, and a high mechanical strength of 57.7 MPa. The C-R-S polymer grout conforms to the Herschel–Bulkley model, and the fitted value of R^2^ is above 0.99. All analyses confirm that the preparation process of C-R-S grouting cementitious material not only substantially improves the utilization rate of red mud, but also provides a theoretical basis for the high-volume application of red mud in the field of grouting.

## 1. Introduction

Lately, the application of deep geotechnical engineering such as mine exploitation, hydroelectric tunnels, and traffic tunnels has attracted much attention [1]. When tunnelling and underground construction projects are faced with unfavorable geological conditions, grouting is the commonly used technique for disaster prevention and mitigation [2]. Grouting material is suitable for a wide range of applications in the reinforcement of mining underground works due to its strength, fluidity, and structural stability [3]. Mudstone cracks are discontinuous surfaces formed by the rupture of rock under stress. Weakening or failure of the anchoring structure may be triggered when groundwater seeps into the mudstone cracks, which is often a significant contributing factor in the failure of support structures. For instance, loosening of the anchoring bolts in the muddy peripheral rock usually triggers a tunnel collapse, thus causing incalculable casualties and financial losses [4,5]. In actual geotechnical engineering, the load-bearing structure of the broken surrounding rock can be restored by grouting in the cracks, thus effectively improving the bearing capacity and integrity of the surrounding rock. The grouting material is one of the cores of grouting reinforcement technology, and its mechanical properties and fluidity determine the feasibility of grouting reinforcement projects [6]. Currently, among the grouting materials that have been developed, organic chemical-based grouting materials are unsuitable for large-scale construction due to the exorbitant cost. In contrast, inorganic grouting materials and cement-based materials are currently the most widely used grouting materials [7,8].

Furthermore, a variety of special cement-based grouting materials with excellent performance have been developed in the existing market [9,10,11], which are roughly divided into three categories: cement grouting materials, cement water glass, and chemical grouting materials. The conventional cement grouting materials are limited to the maintenance of cracks with a width of 0.1 mm, that is, close macroscopic cracks in the rock mass [12]. Moreover, the wide application of cement grouting materials leads to massive CO_2_ emissions, with the cement industry contributing around 8 percent of annual global emissions [13]. Since the European Union released the “European Green Deal” in 2019, taking the lead in proposing targets and related policies to achieve carbon neutrality, countries such as the UK, Sweden, China, South Korea, and the US have also proposed “carbon neutrality” targets [14]. Not only do cementitious grouting materials consume large amounts of resources and energy, but they also have obvious drawbacks, including long setting times and poor durability [15,16,17]. To reduce the burden on the planet and respond to the Chinese government’s “Peak Carbon Neutral” strategy, there is an urgent need for the synthesis of green and low-carbon grouting materials.

Geopolymers are widely utilized in numerous fields, such as road base and construction engineering, due to their low density, low thermal conductivity, and low temperature expansion coefficient. Currently, the use of geopolymers to explore novel grouting materials has become a common trend [18]. Taher et al. [19] investigated the use of bottom ash to stabilize chalky soils and revealed that the geopolymers have the potential to replace silicate cements in grouting materials based on the advantages of environmental friendliness, strength, durability, and excellent chemical properties. Gullu et al. [20] found that FA and geopolymer aggregates can be used as grouting materials to replace natural cement. Geopolymers have different raw materials and manufacturing processes, each with significant advantages and disadvantages, to suit diverse applications. In general, the raw materials applied in geopolymers mainly include industrial by-products containing large amounts of soluble silica and alumina, and the correct choice of raw material type is crucial to the performance of novel grouting materials. The purpose is to find a potential geopolymer as a grouting material alternative and to fulfil the requirement that the material grout can pass through narrow gaps. Meanwhile, underground geotechnical engineering requires that grouting materials exhibit better early strength characteristics and the continued growth of strength in the later stages. The activation degree of ground granulated blast furnace slag (GGBS) is higher than that of other industrial by-products, and after alkali activation, it can significantly improve the strength of the solidification. It is reported that the one of mineral components of GGBS is nano-silica [21,22], and as one of the most effective and finely dispersed mineral additives, nano-silica is presented as amorphous silica in the form of spherical particles; in addition, its considerable specific surface area and amorphous structure confer a high volcanic ash activity. The use of nano-silica as a mineral additive proves to be a promising method for the preparation of high-performance grouting materials, thus contributing to the process of major innovations and advances in modern concrete technology [23]. Calcium content can influence the development of geopolymer strength, and an appropriate calcium content positively affects the compressive strength and microstructure of geopolymers. Zhao et al. [24] explored the impact of the form of calcium on the existence of geopolymer gel products in fly ash-based geopolymers, and it was revealed that the microstructure of the geopolymer became more cohesive and the mechanical properties of the geopolymer improved with an increase in Ca^2+^ content. Li et al. [25] prepared a red mud–blast furnace slag-based grouting material using ultrafine red mud and analyzed the effect of ultrafine red mud on the slurry. It is observed that the addition of red mud extends the setting time of a slag-based mass polymer slurry, and the activation degree of red mud is lower than that of slag, which can be continuously activated in an alkaline environment to increase the strength, while the unreacted red mud particles can fill up the pores of the solidified body to optimize the load-bearing capacity. Therefore, the use of red mud in the preparation of grouting materials not only minimizes the pollution of the alumina industry to the environment, but also reduce the consumption of cement in the construction industry, which inhibits carbon emissions, thus achieving the national strategic goal of “peak carbon” as early as possible [26].

Based on the above considerations, ground granulated blast furnace slag (GGBS), red mud (RM), and ultrafine calcium oxide were utilized as the raw materials for the preparation of composite grouting materials, C-R-S (calcium oxide–red mud–slag). Furthermore, the effect of RM dosage on the fluidity properties, hydration properties, corrosion resistance, and mechanical properties of the grouting materials was investigated in detail. Fourier transform infrared spectroscopy (FT-IR), X-ray diffraction (XRD), TG and derivative thermogravimetry (DTG), scanning electron microscopy (SEM), and a fluidity mechanism were employed to explore the inherent mechanistic issues. The results show that all the properties of the composite grouting material meet the requirements of underground geotechnical grouting projects; in addition, it realizes the consumption of a large number of industrial by-products, thus achieving low-carbon emission reduction and contributing to the development of alkali activation techniques using cementless special grouting materials.

## 2. Materials and Methods

### 2.1. Materials

**Figure 1:**
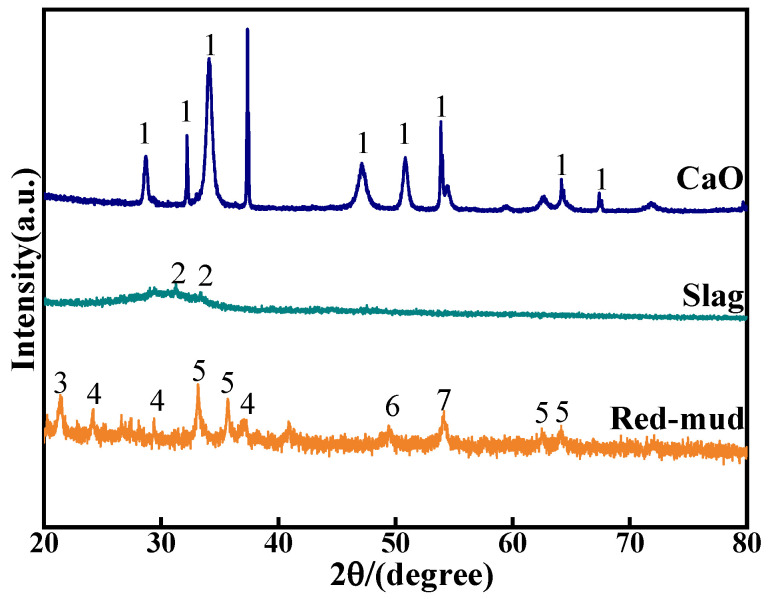
XRD pattern of raw materials (1: CaO, 2: quartz, 3: calcium nepheline, 4: FeO(OH), 5: hematite, 6: mullite, and 7: boehmite).

The red mud used in this experiment was obtained from the Guangxi Fangchenggang Huasheng New Material Company (Fangchenggang, China) from the Bayer method of alumina production, with a pH value of 10.36 and a specific surface area of 23.1 m^2^ g^−1^. The ground granulated blast furnace slag (GGBS), which was purchased from Gongyi Longze Water Purification Materials Co., Gongyi, China, is the S95 standard slag powder. The main chemical compositions and XRD patterns of RM, GGBS, and ultrafine calcium oxide are illustrated in Table 1 and Figure 1, and they reveal that the mineral composition of the GGBS powder is not obvious and show the overall composition of the material in the glassy state. Whereas the phase composition has an important influence on the volcanic ash activity of mineral powders (GGBS), many studies have shown that the glassy state (amorphous phase) is the more active phase in auxiliary cementitious materials (e.g., GGBS), suggesting that the material is potentially volcanic ash active. Here, volcanic ash activity refers to the reactive silica–aluminum substances produced by the decomposition of solid waste in an alkaline environment. Therefore, the higher the content of soluble Si and Al in the ore powder, the higher the degree of hydration reaction it can undergo and the more hydration products it can produce. The exciter is a mixed solution of water glass and NaOH, with the following chemical composition of 8.73% Na_2_O, 27.90% SiO_2_, and 57.60% H_2_O. And the ratio of sodium silicate to sodium silicate (SiO_2_/Na_2_O) is 3.20, the light white sodium silicate solution is easily soluble in water, and the purity of the NaOH is 96% (AR) from Guangxi Xilong Chemical Co. (Yulin, China). The test water is deionized water from the laboratory.

### 2.2. Materials Preparation

#### 2.2.1. Pre-Treatment of Raw Materials

Firstly, the red mud and calcium oxide powder are dried at 80 °C. Subsequently, the dried red mud powder is subjected to ball milling by a cement ball mill tester for 2 h and then passed through a 200-mesh screen to obtain the pre-treated red mud powder. Similarly, the dried calcium oxide powder is ground in a high-energy ball mill for 8 h and then passed through a 2000-mesh screen to obtain ultrafine calcium oxide powder.

#### 2.2.2. Preparation of C-R-S Geopolymer Mortar

The specific synthesis parameters are as follows: the water glass is a sodium silicate solution with an initial modulus (silica–sodium ratio) of 3.20, the silica–sodium ratio of 1.25 adjusted by adding NaOH (sodium hydroxide), as well as a sodium silicate content of 10%; the water–cement ratio is 0.45; the dosage of red mud is 20%, 40%, 60%, and 80%; and the additions of slag are 75%, 55%, 35%, and 15%, respectively. In addition, the addition of ultrafine calcium oxide is 5%, with a maintenance temperature of 20 °C and a humidity of 90%. Finally, the mixing, molding, and the test methods of C-R-S geopolymer grouting materials refer to GB/T 17671-2021 “Cementitious Sand Strength Test Methods” for experiments.

### 2.3. Testing Items and Methods

#### 2.3.1. Compressive Strength

The compressive strength of the cementitious materials was determined in accordance with the Chinese standard GB/T 17671-1999, the specimen size of the cementitious materials was 40 × 40 × 160 mm, and the cured specimens were tested for compressive strength on the 3rd, 7th, and 28th days after curing, respectively. At each curing age, the average value of the three specimens measured was taken as the determined strength.

#### 2.3.2. Fluidity Analysis

The fluidity of the cement paste was determined with reference to the Chinese building materials industry standard (JC/T0508-2005) ‘Cement Slurry Flow Determination Method’. A 60 cm × 60 cm glass plate was prepared, with a wet towel to wipe the surface of the glass plate. The slurry mixed in the cement mortar mixer was uniformly injected into a 60 mm × 60 mm × 36 mm truncated cone mold, the surface was scraped flat with a spatula immediately after filling, the truncated cone mold was lifted, and then the flow diameter was measured with a steel ruler after 30 s to derive the fluidity of the slurry.

#### 2.3.3. Setting Time Analysis

According to GB/T 1346-2011 “Standard Consistency Water Consumption, Setting Time, Stability Test Method Standard of Cement”, the specimen will be taken out of the maintenance for a period of time, and the test needle will be vertically immersed into the mortar body to be tested by Vickers instrument, and it will be proved to reach the initial solidification time when the test needle is immersed into the floor at a distance of 4 mm ± 1 mm. When the circular test needle sinks into the sample 0.5 mm, it is proved that the final setting time is reached.

#### 2.3.4. Bleeding Rate Analysis

A 100 mL measuring cylinder was prepared, the stirred slurry was quickly injected into the inside of the cylinder and accurately reached the 100 mL scale line, and the cylinder was covered with a lid and left; every 15 min, a rubber-tipped burette was used to partially suction out the water secretion, and suction secretion was cushioned on one side of the volumetric cylinder, so that secretion was concentrated, and the pipette suctioned out the secretion injected into the cylinder with a stopper to record the cumulative amount of water secretion each time. After each suction, the volumetric cylinder was then gently flattened.

#### 2.3.5. Characterization of C-R-S Geopolymer

The particle size distribution of the raw materials was analyzed using a nanoparticle size and Zeta potential analyzer (NaniZS, Malvern, UK). The dried powder samples were dispersed in high-purity water at a ratio of 1:100, and ultrasound was used to make the dispersion homogeneous, followed by particle size testing. An X-ray diffraction analyzer (XRD, X’Pert PRO, Almelo, The Netherlands) was used to analyze the physical phases of the raw materials and hydration products; an S-4800 field emission scanning electron microscope (SEM, S-4800, Hitachi Works, Ltd., Tokyo, Japan) was used to observe the morphology of the raw materials and geopolymer; a three-dimensional CT (3D-CT Xradia Crystal CT analyzer, Carl Zeiss AG, Jena, Germany) was used to observe the internal defects of the geopolymer; and Fourier Transform Infrared Spectroscopy (FTIR, Nexus 470, Nicolet Ltd., Madison, WI, USA) was used to scan the chemical bonds of the hydration products. Preliminary analysis of the chemical element concentrations of the polymerization and hydration products of binary cementitious materials was in conjunction with elemental mapping (EDS). Heavy metal concentrations in the leachate were measured by Inductively Coupled Plasma Emission Spectroscopy (Optima 8000 (ICP-OES), Perkin Elmer, Waltham, MA, USA).

## 3. Results and Discussion

### 3.1. Influence of Each Parameter on Material Properties

#### 3.1.1. Effect of Red Mud Additions

**Figure 2:**
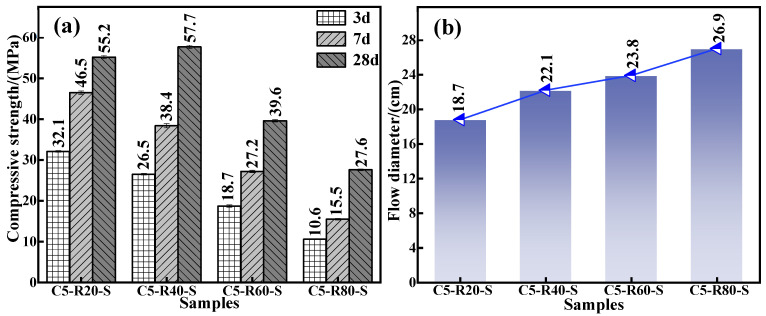
Effect of red mud addition on compressive strength (**a**) and fluidity (**b**) of C-R-S grouting material (in Cm-Rn-S, m and n are the additions of calcium oxide and red mud, respectively).

To inflate the use amount of red mud and meet the requirements of mechanical properties and flowability of the grouting material in practical application, the effect of red mud addition on the mechanical properties and flowability of the C-R-S geopolymer grouting material was carefully investigated. The experimental conditions were as follows: a series of grouting materials (C5-R20-S, C5-R40-S, C5-R60-S, and C5-R80-S) were prepared with a calcium oxide dosage of 5%, a silica–sodium ratio of 1.2, a water glass dosage of 20%, a water–binder ratio of 0.5, and red mud additions of 20%, 40%, 60%, and 80%, respectively. The mechanical properties of the diverse specimen are shown in Figure 2a; in the pre-conservation period (3d and 7d), the compressive strength of the grouting material exhibits a decrease with the red mud addition increasement. The rationality of this trend’s appearance is the fact that the boost in the red mud addition induces an amount reduction in mineral powder in the material and the active Si and Al components in its reaction system, as well as retards the geopolymerization reaction. Consequently, it brings about a decline in the mechanical properties.

Nevertheless, the compressive strength of the C-R-S geopolymer after 28 days of maintenance shows an increasing and then decreasing trend with the changes in red mud addition from 20% to 80%. This is due to the presence of an aluminosilicate component in the red mud, which can participate in geopolymerization and still leach some Si^4+^ and Al^3+^, albeit with low reactivity [27]. After this, appropriately increasing the red mud addition amount then arouses a promoted polymerization reaction between Si^4+^ and Al^3+^ inside the material, facilitating the formation of Si-O-Si (calcium hydrate silicate) and Si-O-Al (aluminum hydrate silicate) structures and, furthermore, enhancing the compressive strength of C-R-S grouting material; more precisely, the compressive strength of C5-R40-S is 4% higher than that of C5-R20-S. For all this, the volcanic ash activity contained in red mud is much lower than that of mineral powder because the red mud obtained by the Bayer method contains half or more the amount of hematite, and its silica–aluminum component content is relatively low. As a result, continuous augmentation of the red mud addition amount will gradually decay the silica–aluminum component in the reaction system and instead escalate the concentration of the iron-containing component, which makes the C-R-S grouting material have fewer active substances during the polymerization process [28]. In addition, the related iron-containing component in the red mud is the essential factor weakening the compression-resistant properties of the cementitious material, so the continuous elevation of the addition amount of red mud will significantly undermine its mechanical properties. From Figure 2b, it can be observed that the flowability of the grouting material exhibits a significant improvement for the increment of red mud addition, and the flowability increases from 18.7 cm to 26.9 cm. The larger the amount of red mud dosed, the fewer active components in the system, thus slowing the depolymerization and condensation reactions in the red mud and the slag, as well as extending the solidification time, both of which lead to the increase in the flowability. Combining the above experimental results, on the basis of consuming as much red mud as possible to meet the requirements of the material’s mechanics and fluidity in practical application, the amount of red mud added in the subsequent experiments was taken as 40%.

#### 3.1.2. Effect of Calcium Oxide Additions

**Figure 3:**
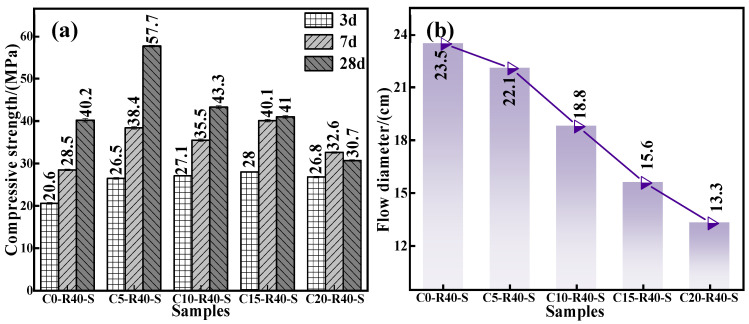
Effect of calcium oxide addition on compressive strength (**a**) and fluidity (**b**) of C-R-S grouting material.

To gain insight into effect of calcium oxide addition on the mechanical properties and flowability of the C-R-S geopolymer, a series of grouting materials (C0-R40-S, C5-R40-S, C10-R40-S, C15-R40-S, and C20-R40-S) were synthesized with conditions of red mud addition of 40%, silica to sodium ratio of 1.2, water glass dosage of 20%, water–binder ratio of 0.5, and calcium oxide additions of 0%, 5%, 10%, 15%, and 20%, respectively. Figure 3a illustrates the compressive strength of the C-R-S geopolymers with diverse calcium oxide additions, and it demonstrates that the compressive strength of the C-R-S grouting material firstly shows a positive and then a negative correlation with the amount of ultrafine calcium oxide added. When the addition of ultrafine calcium oxide is 5%, it can be obviously observed that the moderate addition of calcium oxide improves its 28 d mechanical properties by more than 30% compared to the calcium oxide addition of 0%, from 40.2 MPa to 57.7 MPa. In the actual hydration reaction process, the calcium component promotes the hydration reaction of silica–alumina, leading to the excellent mechanical strength of the gel system [29]. What is more, the Ca^2+^ in calcium oxide can participate in the copolymerization reaction under the action of the initiator to form a copolymer gel with the structure of N-(C)-S-A-H [24], which optimizes the pore structure of the matrix and makes it more compact, so that its mechanical strength is improved [18]. When the dosage of calcium oxide is higher than 5%, the calcium oxide is not completely hydrated into C-S-H gel in the process of geopolymerization, and the excess is in the form of calcium hydroxide, so in the pre-maintenance period (3d), the compressive properties of the grouting material basically remain unchanged. However, in the late maintenance process (7 d and 28 d), CO_2_ in the air enters into the system and reacts with calcium hydroxide to generate calcium carbonate, and the geopolymer shows the phenomenon of carbonation and contraction or even cracking, leading to a sharp decrease in mechanical properties. The compressive strength at 7 d decreased from 38.4 MPa to 32.6 MPa (15.1% decrease), and that at 28 d decreased from 57.7 MPa to 30.7 MPa (46.79% decrease).

From Figure 3b, it can be seen that the flowability of the grouting material presents an inversely proportional trend to the calcium oxide addition due to the fact that Ca^2+^ in calcium oxide promotes the hydration reaction of silica–alumina, which accelerates the reaction of silica–alumina in the system and shortens the coagulation time of the geopolymer, and therefore eroding the flowability from 25.3 cm to 13.3 cm. Combining the above experimental data, taking the mechanical properties and the flowability into consideration, we selected the calcium oxide addition as 5% in the subsequent experiments.

#### 3.1.3. Effect of Silicate to Sodium Ratios

**Figure 4:**
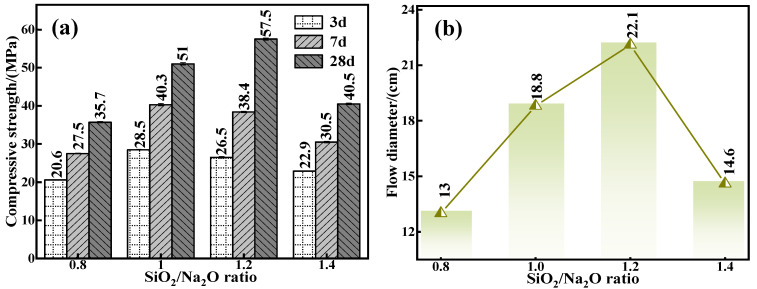
Effect of water glass modulus on mechanical properties (**a**) and flowability (**b**) of C-R-S grouting materials.

To further scrutinize the intrinsic influence of the silica to sodium ratio on the mechanical properties and flowability of the C-R-S geopolymers, actual experiment conditions of a calcium oxide addition of 5%, red mud addition of 40%, water glass dosage of 20%, water–binder ratio of 0.5, silicate to sodium ratios of 0.8, 1.0, 1.2, and 1.4, respectively, were chosen to prepare a series of grouting materials (C5-R40-S-x; x is the ratio of sodium–silica, i.e., the molar ratio of SiO_2_/Na_2_O). The mechanical properties of individual grouting materials (Figure 4a) were noted, and when the water–cement ratio is increased from 0.8 to 1.4, the 28 d mechanical properties increase from 35.7 MPa (C5-R40-S-0.8) to 57.7 MPa (C5-R40-S-1.2) by 61.62%, and then decrease to 40.5 MPa (C5-R40-S-1.4) by 29.81%; additionally, the overall trend is upward and then downward. The reason is that when the silica to sodium ratio is low, the alkalinity of alkaline exciters is high, and the excessive OH^−^ in the system will destroy the chemical equilibrium of the geopolymerization reaction [30], and when the ratio of silica to sodium is optimized to 1.2, the moderate alkalinity in the reaction system generates a good deal of SiO_3_^2−^ and AlO_2_^−^ in the hydration reaction. Likewise, with the hydration time, a further polymerization reaction occurs at the interface between the monomer and dimer, which forms a multimeric three-dimensional reticulated geopolymer gel [31]. Subsequently, the internal structure of the grouting material is gradually stabilized, thus enhancing its mechanical properties. Meanwhile, with the sodium silicate ratio as high as 1.4, in polymerization reaction systems, too low an alkalinity can cause an incomplete polymerization reaction, leading to a conspicuous reduction in its mechanical properties instead.

The results of the flowability of diverse C5-R40-Ss are demonstrated in Figure 4b, and it shows that, as with the mechanical properties, its flowability with the rise in the Si/Na ratio shows the phenomenon of first enhancing and then diminishing. The alkalinity in the system is inversely proportional to the silica–sodium ratio, and the high alkalinity caused by the low silica–sodium ratio expedites the polymerization reaction, forming a three-dimensional mesh structure in a relatively short period of time, with the consequent restriction of the flowability. Therefore, within a certain range (0.8 and 1.0), the lower the ratio of sodium silicate, the feebler the flowability. However, the high ratio of sodium silicate of 1.2 brings a too low alkalinity, which can cause an incomplete polymerization reaction, leading to a conspicuous reduction in the matieral’s mechanical properties instead. Together with the above all results, the ratio of sodium silicate was optimized to 1.2 in the later experiments.

#### 3.1.4. Effect of Water Glass Dosage

**Figure 5:**
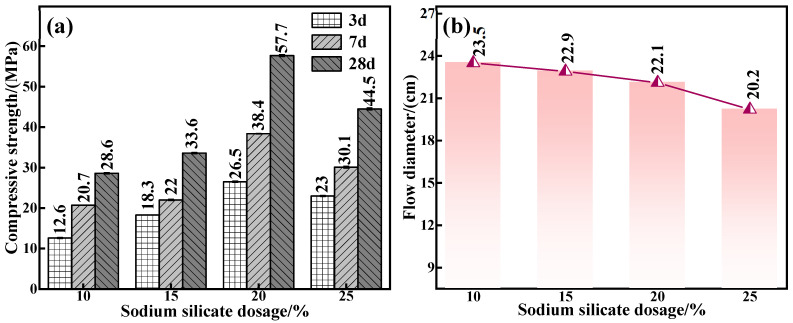
Effect of water glass dosage on mechanical properties (**a**) and flowability (**b**) of C-R-S grouting materials.

The endogenous connection between water glass dosage and the mechanical properties and fluidity of C-R-S geopolymers was further corroborated. The experimental conditions were as follows: a calcium oxide addition of 5%, red mud addition of 40%, silica–sodium ratio of 1.2, water–binder ratio of 0.5, and water glass dosage of 10%, 15%, 20%, and 25% were applied to prepare a series of grouting materials (C5-R40-S-y, with y being the water glass dosage). Figure 5a reveals that when the water glass dosage is in the interval of 10–20%, the mechanical properties of the grouting material show an upward trend, and the maximum compressive strength is up to 57.7 MPa, and the increase in the compressive strength of the C5-R40-S-20% compared with that of C5-R40-S-10% addition amounts to 50.4%. In general, Na_2_O and SiO_2_ are involved in the depolymerization and condensation reaction [27] in the process of the red mud hydration reaction. Under a too low water glass dosage, the concentration of Na_2_O and the content of free SiO_2_ in the system are low, resulting in an insignificant polymerization reaction and poor mechanical properties. However, with the amount of water glass increasing to 25%, the added OH^−^ concentration is too high, which disrupts the reaction equilibrium within the system and therefore leads to the deterioration of its mechanical properties.

The related results of the flowability of the various C5-R40-S-ys specimen blocks are presented in Figure 5b; as can be seen, flowability decreases (from 23.5 cm to 20.2 cm) with the rise in water glass dosage, which is attributed to the fact that in the process of the red mud hydration reaction, the high concentration of Na_2_O and SiO_2_ (under the lower water glass dosage) can promote the depolymerization and condensation reaction of the grouting material [27], thus generating a flowability deterioration. Meanwhile, the enhanced water glass dosage in the reaction system causes a gradual increase in free OH^−^ and SiO_2_, which leads to the generation of geopolymer chain structures such as C-S-H\C-S(A)-H with calcium-based components [27]. From the above results, it can be inferred that the water glass dosage should be selected as 20% in the subsequent experiments.

#### 3.1.5. Effect of Water–Binder Ratio

As with these previous experimental parameters above, the following experimental conditions were used: a calcium oxide addition of 5%, red mud addition of 40%, silica-sodium ratio of 1.0, water glass dosage of 20%, and water–binder ratios of 0.5, 0.6, 0.7, and 0.8 were utilized to prepare a series of grouting materials (C5-R40-S-z, with x being the water–gel ratio, i.e., the mass ratio of water to sodium silicate), and investigate the effect of the water–binder ratio on the C-R-S grouting materials’ properties.

**Figure 6:**
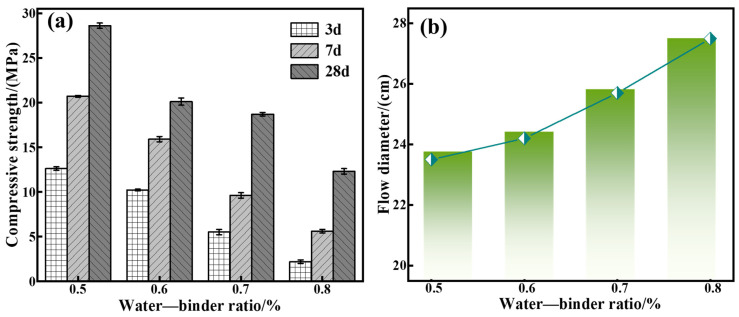
Effect of water–binder ratio on mechanical properties (**a**) and flowability (**b**) of C-R-S grouting materials.

Figure 6a shows that when the water–binder ratio increases from 0.5 to 0.8, the compressive strength after maintenance for 28 d decreases from 57.7 MPa to 16.6 MPa, and Figure 6b illustrates that the increasing water–binder ratio is conducive to a flowability increment. The reason is that the lower water–binder ratio, the larger the OH^−^ concentration in the system, which makes the polymerization reaction in the system accelerate rapidly. On the contrary, when the water–binder ratio increases, the polymerization reaction is slowed down, and the reaction is insufficient. And it produces a destabilization of the grouting material structure, which weakens the mechanical properties but enhances its flowability. According to the related trends of the mechanical properties and fluidity analysis, it can be seen that the effect of the water–gel ratio on the mechanical properties and fluidity of the material is opposite, but the change interval of fluidity is fine (23.5 cm~27.4 cm, just 16.59%), while the effect on the mechanical properties is larger, and the compressive strength decreases from 57.7 MPa to 16.6 MPa (up to 71.23%); therefore, in the practical application, the effect of the water–binder ratio on the compressive strength should be taken more into consideration and, as a result, the water–binder ratio should be selected as 0.5 in subsequent experiments.

### 3.2. Performance Analysis of C-R-S Geopolymer Grouts

#### 3.2.1. Setting Time Analysis of C-R-S Geopolymer Grouts

**Figure 7:**
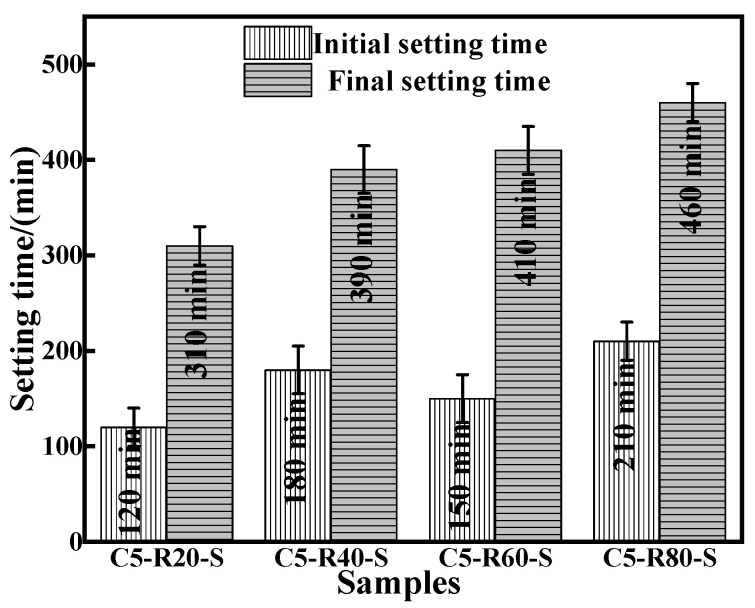
Comparison of initial and final setting time of diverse synthesized C-R-S grouting materials.

The initial and final setting times of the C-R-S geopolymer grouts are shown in Figure 7. With the increase in red mud content, the initial solidification time of the geopolymer slurry presents the phenomenon of first increasing, then decreasing, and then increasing, and this is due to the red mud content of 20% and the greater slag content; the hydration reaction is intense and consumes a lot of water, so the geopolymer slurry solidification time is shortened. With a red mud content of 60%, the red mud itself consumes a lot of water to make the early condensation time faster, but the hydration reaction is not terminated, so the final condensation time slower; with a red mud content of 40%, the initial condensation time slower. And the final coagulation time is prolonged with the increase in red mud content, which is related to the degree of the hydration reaction of the system itself, and as the hydration reaction proceeds, the red mud is also gradually involved in the reaction.

#### 3.2.2. Analysis of the Water Secretion of C-R-S Geopolymer Grouts

**Figure 8:**
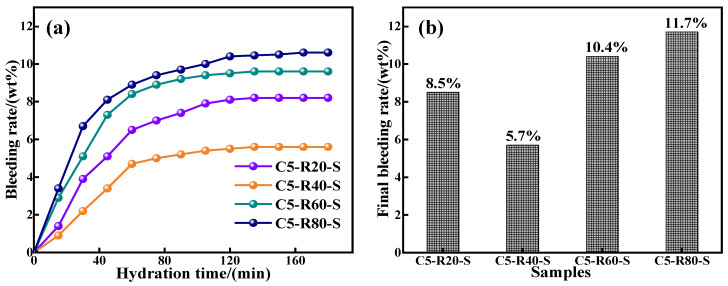
Results of bleeding rate (**a**) and final bleeding rate (**b**) of C-R-S grouting materials.

The water seepage property has a significant impact on the solidity of the grout, which is directly related to the reactivity of the raw material, specific surface area, and particle size. The water secretion properties are shown in Figure 8. Except for the red mud content of 40%, the water seepage performance of the C-R-S geopolymer slurry increases gradually with the increase in red mud content, and increases gradually with the increase in hydration time, and finally tends to level off. This is because the increase in red mud slows down the hydration reaction in the system, the gelling properties of red mud are lower than for slag, and the increase in red mud reduces the stability of the hydration system; although the specific surface area and particle size of red mud are higher than those of slag, with the hydration reaction, the water in the system is gradually secreted. When the content of red mud is 40%, the hydration reaction system is the most stable, and fewer free water molecules are inside the slurry, part of which is involved in the hydration reaction and part of which is absorbed by the red mud. As the hydration reaction proceeds, the water molecules absorbed by the red mud continue to participate in the hydration reaction, and only a small amount of water is seeped out, and the slurry hydration reaction of the C-R-S geopolymer injection slurry is always in equilibrium. The lowest water secretion rate was 5.7% at 40% red mud content.

#### 3.2.3. Fluidity Properties of C-R-S Grouting Materials

**Figure 9:**
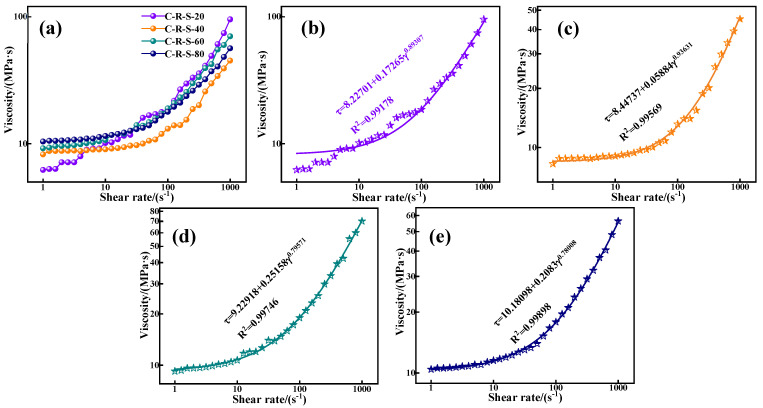
The effect of shear rate on shear stress of C-R-S grouting materials (**a**) and the fitted plots of C5-R20-S (**b**), C5-R40-S (**c**), C5-R60-S (**d**), and C5-R80-S (**e**), separately.

In the grouting project, the rheological properties of the grouting material determine the pumping distance of the grout; they are determined by the granular properties of the material and its hydration process, and the granular properties of the material depend on the particle size of the material, the specific surface area, the degree of activity, and the affinity for water. Figure 9a depicts the relationship between shear rate and shear stress between C-R-S geopolymer grouts with different mixing ratios, and deeply analyzes the effect of different red mud contents on the rheological properties of grouting materials. As can be seen from the figure, except for the red mud addition of 40%, the reduction in red mud content has a positive influence on the rheology and flow of the C-R-S geopolymer slurry, and the shear stress of the material decreases gradually with the increase in red mud content. This may be because the activity of red mud is much lower than that caused by slag, and although the red mud specific surface area is larger, the particle size is smaller, and the water absorption is stronger, but the degree of reaction in the hydration process is lower; with the increase in the shear rate, the slag plays a decisive role in the hydration process when the slurry is fully reacted, and the fact that the high content of red mud in the low shear rate exhibits a stronger shear stress is precisely due to the water absorption of the red mud.

The relationship between the shear rate and shear stress of the grouting material presented in Figure 9a by the Herschel–Buckley model [25], which describes a typical non-Newtonian fluid without time dependence in its form of expression, and is between the Bingham and the power law model at a low shear rate, synthesizing the characteristics of the Bingham model and the power law model, which is of great significance in underground grouting. Its model expression is as follows:

(1) $$\tau =\tau_{0}+K\gamma^{n}$$

where *τ* is the shear stress, *τ*_0_ is the yield stress, *K* is the consistency factor, γ is the shear rate, and n is the flow coefficient. As can be seen from Table 2 and Figure 9b–e, the C-R-S geopolymer grouts all conform to the expression of the Herschel–Buckley model, and their fitted values of R^2^ are all above 0.99. The C-R-S geopolymer grout with 40% hematite content exhibits a lower shear stress at lower shear rates and exhibits a greater increase in shear stress when the shear rate is higher; the consistency coefficient (*K*) of the C-R-S geopolymer slurry with 40% red mud content is small, 0.05884, and the flow coefficient (*n*) is large, 0.93631, which proves that the C-R-S geopolymer slurry has good flow characteristics in the pre-metamorphic reaction period, and strong mechanical properties in the late period of the metamorphic reaction.

### 3.3. Structural Characterization Analysis of C-R-S Grouting Materials

#### 3.3.1. SEM, XRD, and FTIR Analysis of C-R-S Grouting Materials

**Figure 10:**
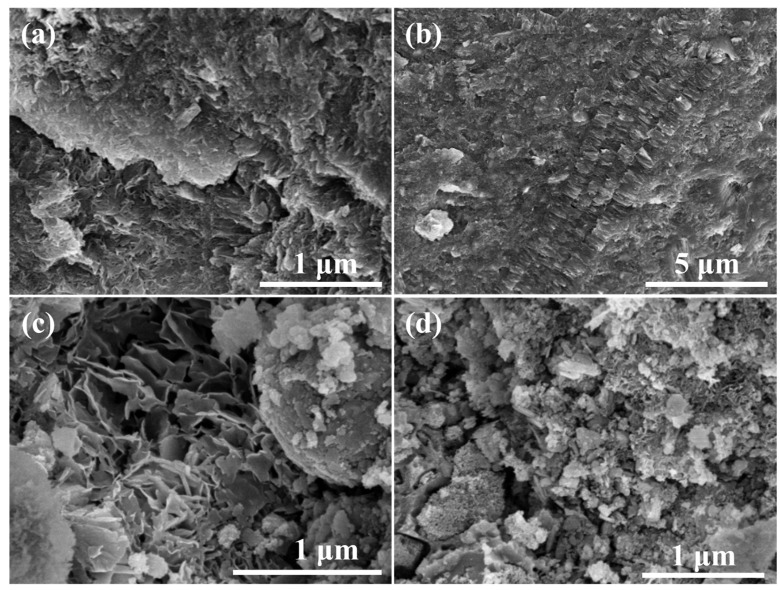
SEM images of hydration products of grouting materials C5-R20-S (**a**), C5-R40-S (**b**), C5-R60-S (**c**), and C5-R80-S (**d**), separately.

Figure 10a shows the SEM image of the specimen with 20% red mud addition, from which it can be observed that the surface flatness is poor, but the overall structure is more compact. Figure 10b presents the SEM image of the specimen with 40% red mud addition, from which it can be observed that most of the C-R-S geopolymer morphology shows a lamellar structure, and the overall structure is more compact. From this figure, it can be observed that regarding the microscopic morphology of C-R-S during the hydration process, the hydration process formed a geopolymer skeleton structure such as C-S-H, and the unreacted red mud particles were solidified in the lamellar structure. Figure 10c illustrates the SEM image of the test block with 60% red mud addition, from which it can be observed that the surface of the C-R-S geopolymer is uneven, and the middle part is still formed in an irregular lamellar combination. Figure 10d reveals the SEM image of the test block with 80% red mud addition, from which it can be observed that the surface of the C-R-S geopolymer has a very poor leveling pattern and the structure is very loose.

**Figure 11:**
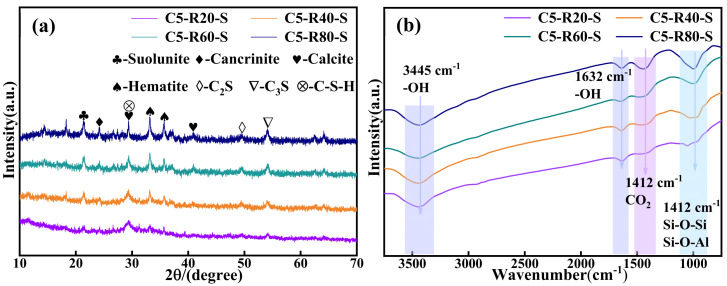
XRD patterns (**a**) and FTIR curves (**b**) of C-R-S grouting materials.

The XRD physical phase analysis of the C-R-S geopolymer is shown in Figure 11a. The C-R-S geopolymer mainly exists in the form of Ca_2_[Si_2_O_5_(OH)_2_]-H_2_O, CaCO_3_, Fe_2_O_3_, KNa_3_[AlSiO_4_], C_2_S, C_3_S, etc., and the diffraction peaks at 20–30° are those of the geopolymer gel C-S-H; the peaks of hematite indicate that a large amount of hematite is not involved in the hydration process in the sample. The presence of calcium hydroxide-based substances was not observed in the XRD analysis, which suggests that the hydration reaction of the ultrafine calcium oxide added during the experiment was relatively complete. The chemical bonding analysis of the C-R-S geopolymer is shown in Figure 11b, with the vibrational peaks of OH- at 3445 cm^−1^ and 1632 cm^−1^; the vibrational peak at 1412 cm^−1^ is the absorption peak of CO_2_, which indicates that substances such as Na_2_CO_3_ or CaCO_3_ were formed by the carbonation reaction, and there are absorption peaks of Si-O-Si or Si-O-Al at 969 cm^−1^, which were formed by the geopolymer polymerization reaction.

#### 3.3.2. Defect Analysis of C-R-S Geopolymer Grouts

**Figure 12:**
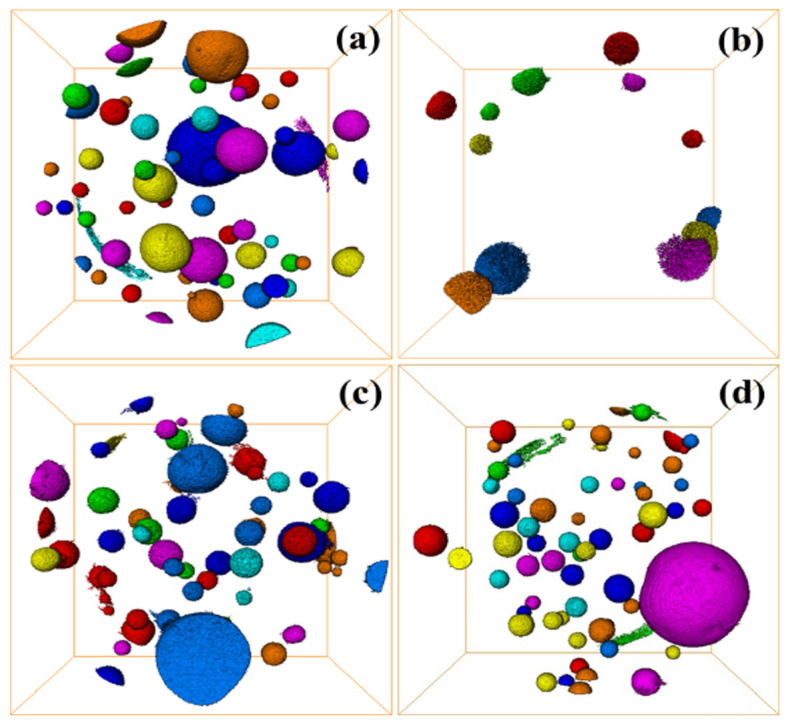
Defect diagram of C-R-S grouting materials: C5-R20-S (**a**), C5-R40-S (**b**), C5-R60-S (**c**), and C5-R80-S (**d**), separately.

The internal defects of grouting materials in underground grouting projects are extremely influential for the mechanical properties of the materials, and the internal cracks and pores of the materials can be characterized by 3D CT. Figure 12a–d represent the internal pores of C-R-S geopolymer slurry when the red mud content is 20%, 40%, 60%, and 80%, respectively, the average volume of the pores is 13,351.0 μm^3^, 5125.76 μm^3^, 8548.92 μm^3^, and 21,641.9 μm^3^, respectively, and the smallest porosity is 152.0 μm^3^. The maximum porosity is 4.02718 × 108 μm^3^. Additionally, the results confirm that the internal porosity of C-R-S geopolymer slurry is the smallest when the content of red mud is 40%, which is matched with its mechanical properties.

#### 3.3.3. Thermal Weight Loss Analysis of C-R-S Geopolymer Slurries

**Figure 13:**
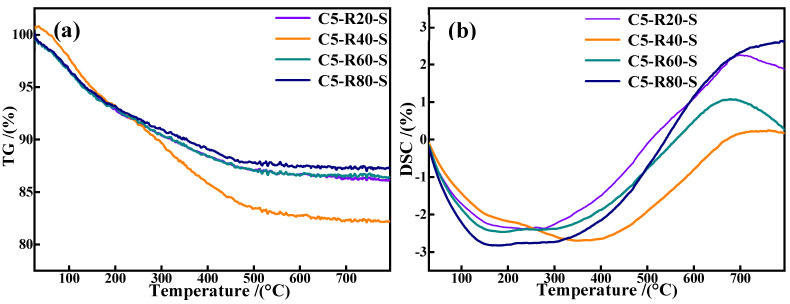
TG curves (**a**) and DSC plots (**b**) of C-R-S grouting materials.

Figure 13 presents the TG-DSC curves of C-R-S geopolymer grouting materials and the plots demonstrate that all the grouting samples continue to lose weight up to 100 °C with a faster rate of weight loss, which is due to the evaporation of free water and hydroxyl groups in the C-R-S geopolymer slurry. When the temperature reaches above 200 °C, the water of crystallization in the C-R-S samples begins to evaporate, and the high temperature leads to the generation of the corresponding oxides in the C-R-S grouting materials, which results in a phase transition with a continuous increase in weight loss and a strong heat absorption peak in the corresponding DSC curve. Meanwhile, the DG plots indicate the continued decomposition of stable chemically bound water in the C-S-H gel structures of the C-R-S grouting materials at a temperature range of 300 °C to 600 °C with a low rate of weight loss [32], and the corresponding DSC curve does not show obvious heat absorption peaks. Comparison of the weight loss of each sample in the temperature interval 300–600 °C reveals that the C5-R40-S grouting material possesses the greatest weight loss, which proves that the C-S-H gel content of C5-R40-S is the highest, thus making the C5-R40-S grouting material the material with the most excellent mechanical properties.

#### 3.3.4. Elemental Analysis of C-R-S Grouting Materials

**Figure 14:**
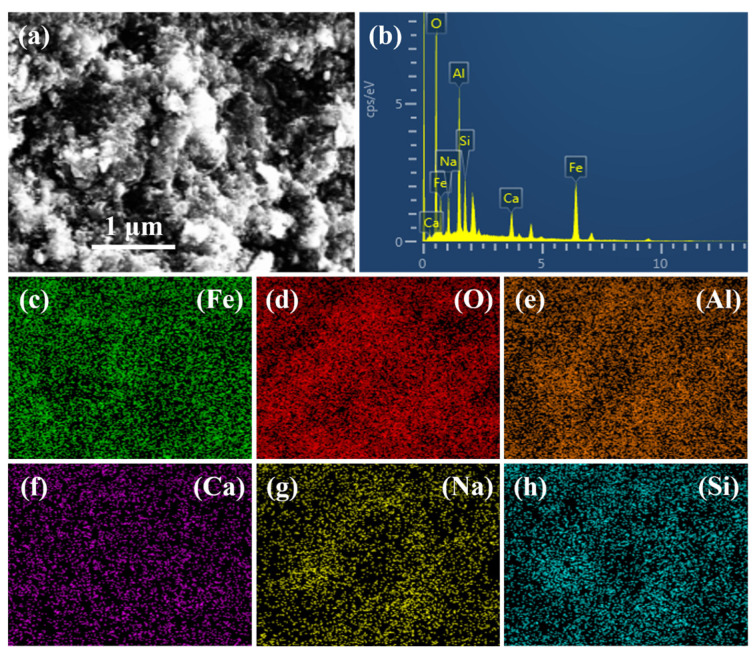
SEM (**a**), energy spectrum (**b**), and element distribution (**c**–**h**) of C-R-S-40 grouting material.

Figure 14 shows the energy spectrum and elemental distribution of the C-R-S geopolymer slurry with 40% red mud content, and the results show that the C-R-S geopolymer slurry is mainly dominated by iron, oxygen, silicon, sodium, calcium, and aluminum; Table 3 shows the atomic percentage of elements in the C-R-S geopolymer slurry with 40% red mud content, of which the iron content is 18.05%, the oxygen content is 55.56%, the silicon content is 4.88%, the sodium content is 5.77%, the calcium content is 2.96, and the aluminum content is 12.79%, proving that the hydration products are dominated by C-S-H, which is consistent with what is shown in the XRD, in which the hydration products contain a large amount of iron from the red mud, and these inert red mud particles are solidified in the C-S-H and C-A-S-H gels, causing a decrease in the mechanical properties.

### 3.4. Leaching of Heavy Metal Ions from Red Mud Base Aggregates

**Figure 15:**
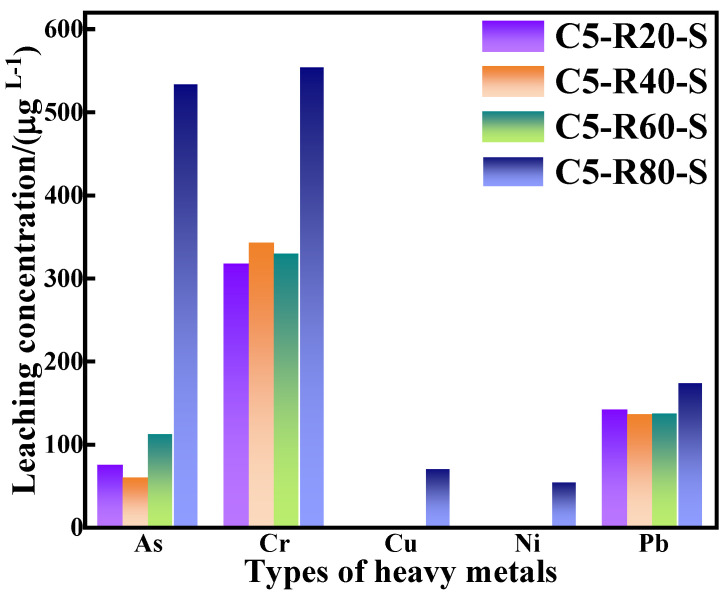
Heavy metal ion leaching from C-R-S geopolymer.

In the grouting project, heavy metal ions of grouting materials can greatly affect the groundwater and underground ecological environment, so it is extremely important to evaluate the leaching of heavy metal ions from grouting materials. The heavy metal ions of the C-R-S grouting materials were measured via leaching experiments, which were based on GB/T 30810-2014 “Determination of Leachable Heavy Metals in Cement Cementitious Sand”. Furthermore, the leached ions were determined by ICP, and the specific experimental results are shown in Figure 15 below.

Combined with the experimental data in Figure 15 and based on the results of GB/T 14848-2017 Groundwater Quality Standards (shown in Table 4), it is revealed that the arsenic (As) concentration in the leachate of the C-R-S grouting materials, except for C5-R80-S, complies with the national Class V groundwater standards. Except for C5-R80-S, the concentration of chromium (Cr) in the leachate of the C-R-S grouting materials meets the national Class V groundwater standards. The concentrations of copper (Cu) and nickel (Ni) in the leachate of all C-R-S grouting materials are in accordance with national Class III groundwater standards; in addition, the concentration of lead (Pb) in the leachate is in accordance with national Class V groundwater standards. The gel product in the C-R-S grouting material exists in a three-dimensional oxide mesh space structure, in which the harmful heavy metals are adsorbed on the calcium sulphoaluminate hydrate (AFt) and C-A-S-H structures as well as on the gelling matrix, which easily realizes the curing effect on the heavy metals, indicating that the prepared grouting material is an environmentally friendly and green material, which does not have any harmful impact on the environment, both in the long term and in the short term.

### 3.5. Analysis of the Strength Formation Mechanism of Grouting Materials

The above results show that the formation of compressive strength and flowability of geopolymer grouting materials is the result of a combination of multiple factors. The formation mechanism of grouting material strength is elaborated from three aspects: the hydration reaction of silicon–aluminum materials, the influence of bentonite on the hydration process of solid waste materials, and the influence of bentonite itself on material strength.

Red mud provides the required silica/aluminum components for the preparation of geopolymers, and the alkaline exciter, by providing an alkaline medium, dissolves the reactive alumina and silica in the red mud, causing the Si-O and Al-O bonds in the red mud to break and form free reactive components, and at the same time, under the action of the water glass, a geopolymerization reaction occurs with the reactive silica and alumina in the GGBS to form an amorphous zeolite-like gel structure. In the middle and late stages of the reaction, part of the incompletely reacted reactive alumina trioxide and silicon oxide react with the calcium hydroxide formed by the dissolution of calcium oxide in the slag to produce C-A-S-H cementitious hydration products. The ultrafine calcium oxide in the system reacts with water in the exciter to generate calcium hydroxide, and the process is accompanied by exothermic effects. The increase in the local temperature of the ultrafine calcium oxide makes the molecules in the reaction system near the ultrafine calcium oxide vibrate and the reaction speeds up. Additionally, due to the formation of calcium hydroxide increasing the alkalinity in the reaction system, it speeds up the depolymerization and re-polymerization reaction of the reaction system and generates [SiO_4_^−^] and [AlO_4_^−^]. Under localized exothermic high-temperature conditions, some of the calcium hydroxide co-participates in the hydration reaction to produce C-S-H gels (i.e., strong base–weak acid salt precipitates insoluble in alkali).

A series of dissolution–condensation reactions of raw materials dissolved in alkaline medium with alkali, alumina, silica, and calcium oxide proceed as follows:

(1) Dissolution stage: Structures such as aluminosilicate in raw materials are dissolved under the action of a strong alkali. A large amount of OH^−^ is dissolved in the alkali exciter, forming alkali metal cations and OH^−^ colloidal solution, in which covalent bonds such as Si-O-Si, Al-O, etc., are broken by hydration under the action of alkali metal cations. Al and Si in the solution increase with the increase in alkalinity of the solution.
  Al_2_O_3_ + 3H_2_O + 2OH^−^ →2[Al(OH)_4_]^−^ SiO_2_ + H_2_O + OH^−^ →[Si(OH)_3_]^−^ SiO_2_ + 2OH^−^ →[SiO_2_(OH)_2_]^2−^
(2) Hydrolysis stage: The silica–aluminum ionophore after fracture interacts with alkali metal ions, OH-, etc., to form monomers such as -Si-O-Na and -Si-O-Ca-OH. Silicic acid generated by hydrolysis is in the gel state and is not soluble in water. Silicic acid in the gel state can react with Ca^2+^ to generate hydrated calcium silicate.
  SiO_3_^2−^ + 2H_2_O→H_2_SiO_3_ + 2OH^−^
(3) Condensation stage: As the dissolution process proceeds, the monomer undergoes a condensation reaction to form an ionic mass, i.e., a gel-like zeolite precursor is formed (in the presence of an alkali exciter).
  (Si_2_O_5_, Al_2_O_2_) + 3nH_2_O→n(OH_)3_–Si–O–Al–(OH)_3_ n(OH)_3_–Si–O–Al–(OH)_3_→(–SiO–O–Al–O–O–)n + 3nH_2_O
(4) Solidification stage: The portion of the zeolite-like precursor formed in the polycondensation stage is subjected to further polycondensation reactions, i.e., the formation of a three-dimensional network structure, which leads to solidification of the material as the network structure continues to expand until it eventually hardens to become a geopolymer (in the presence of an alkali exciter).
  (Si_2_O_5_, Al_2_O_2_) n + 2nSiO_2_ + 4nH_2_O→n(OH)_3_–Si–O–Al–(OH)_2_–O–Si(OH)_3_ n(OH)_3_ –Si–O–Al–(OH)_2_–O–Si(OH)_3_→NaOH(–SiO–O–Al–O–SiO–O–)n + 4nH_2_O

## 4. Conclusions

Ground granulated blast furnace slag (GGBS), red mud (RM), and ultrafine calcium oxide were utilized as the raw materials for the preparation of C-R-S geopolymer cementitious grouting materials, and the effect of RM dosage on the fluidity properties, hydration properties, corrosion resistance, and mechanical properties of the grouting materials was investigated in detail. The results of the experiment are as follows:

(1) (The C5-R40-S grouting material with 40% red mud addition exhibits the best fluidity of 27.5 cm, the lowest water secretion rate of 5.7%, and the highest mechanical properties of 57.7 MPa. This reveals that the C5-R40-S geopolymer slurry has superior fluidity characteristics in the early stage of the hydration reaction and excellent mechanical properties at the later stage, which is completely suitable for extensive utilization in grouting projects.
(2) The microstructure of the C-R-S geopolymer slurry is a layered structure with nano-micropores, and part of the red mud that is not involved in the hydration process can be cured by the C-S-H gels produced by the hydration reaction, thus improving the mechanical properties of the C-R-S geopolymer. The hydration products of the C-R-S geopolymer slurry are mainly calcium carbonate and hydrated calcium silicate, and a large amount of C-S-H geopolymer gel is formed in the process of hydration.
(3) The gel product in the C-R-S grouting material exists in a three-dimensional oxide mesh space structure, in which the harmful heavy metals are adsorbed on the AFt and C-A-S-H structures as well as on the gelling matrix, which easily realizes the curing effect on the heavy metals, except for C5-R80-S; (As) concentration in the leachate of the C-R-S grouting materials complies with the national Class V groundwater standards and the concentration of chromium (Cr) meets Class V standards. The copper (Cu) and nickel (Ni) concentrations of all grouting materials are in accordance with Class III standards; in addition, the concentration of lead (Pb) is in accordance with Class V standards. It is revealed that the synthesis and preparation of grouting materials are in line with China’s low-carbon, green, sustainable industry development.

## Figures and Tables

**Table 1:** Chemical composition of raw materials.

Raw Materials	CaO	SiO_2_	Al_2_O_3_	TiO_2_	Fe_2_O_3_
RM	2.47 ± 0.012	3.66 ± 0.021	16.68 ± 0.054	7.42 ± 0.030	69.77 ± 0.078
GGBS	36.21 ± 0.059	32.50 ± 0.062	15.56 ± 0.042	5.46 ± 0.009	0.6 ± 0.0001
Calcium oxide	98.36 ± 0.012	0.07 ± 0.012	-	-	0.09 ± 0.00002

**Table 2:** The related parameters fitted by Herschel–Buckley model.

Samples	Fitting Equation	R^2^
C5-R20-S	*τ* = 8.22701 + 0.17265γ^0.89307^	0.99178
C5-R40-S	*τ* = 8.44737 + 0.05884γ^0.93631^	0.99569
C5-R60-S	*τ* = 9.22918 + 0.25158γ^0.79571^	0.99746
C5-R80-S	*τ* = 10.18098 + 0.2083γ^0.78008^	0.99898

**Table 3:** The atomic percentage of elements in the C-R-S geopolymer slurry with 40% red mud content

Elements	Fe	O	Al	Ca	Na	Si
wt%	18.05	55.56	12.79	2.96	5.77	4.88

**Table 4:** Criteria values for characteristic pollutants in leachate (mg/L).

Categories	As	Cr	Cu	Ni	Pb	Reference Standard
I	≤0.001	≤0.005	≤0.010	≤0.002	≤0.005	«Groundwater Quality Standard» (GB/T 14848-2017)
II	≤0.001	≤0.010	≤0.050	≤0.002	≤0.005	
III	≤0.010	≤0.050	≤1.000	≤0.020	≤0.010	
IV	≤0.050	≤0.100	≤1.500	≤0.100	≤0.100	
V	>0.050	>0.100	>1.500	>0.100	>0.100	

## Data Availability

The original contributions presented in the study are included in the article, further inquiries can be directed to the corresponding author.

## References

[1] Xu Y., Zhang H., Yu X., Qian Y. (2024). Development and evaluation of physical and mechanical properties of alkali-activated multi-component composite grouting materials. Constr. Build. Mater..

[2] Güllü H., Agha A.A. (2021). The rheological, fresh and strength effects of cold-bonded geopolymer made with metakaolin and slag for grouting. Constr. Build. Mater..

[3] Duan D., Wu H., Wei F., Song H., Ma Z., Chen Z., Cheng F. (2023). Preparation, characterization, and rheological analysis of eco-friendly geopolymer grouting cementitious materials based on industrial solid wastes. J. Build. Eng..

[4] Zhang F.S., Wang X.H., Tang M.R. (2021). Oil well construction engineering-drilling, oil well completion. Rock Mech. Rock Eng..

[5] Qiu X., Fan X.-M., Xu H., Li L., Jiang H.-B., Chen C.-R. (2023). Corrosion characteristics of low-carbon steel anchor bolts in a carbonaceous mudstone environment. J. Cent. South Univ..

[6] Li X., Feng X., Zhou Y., Yang C., Liu X. (2023). Formulation and properties of a new cleaner double liquid alkali-activated grouting material. J. Clean. Prod..

[7] Li S.C., Zhang J., Li Z.F., Gao Y., Qi Y., Li H., Zhang Q. (2019). Investigation and practical application of a new cementitious anti-washout grouting material. Constr. Build. Mater..

[8] Wu T.H., Gao Y.T., Zhou Y. (2022). Application of a novel grouting material for prereinforcement of shield tunnelling adjacent to existing piles in a soft soil area. Tunn. Undergr. Space Technol..

[9] Cui W., Huang J.Y., Song H.F., Xiao M. (2017). Development of two new anti-washout grouting materials using multi-way ANOVA in conjunction with grey relational analysis. Constr. Build. Mater..

[10] Jiang X., Zhu H.H., Yan Z.G., Zhang F., Ye F., Li P., Zhang X., Dai Z., Bai Y., Huang B. (2023). A state-of-art review on development and progress of backfill grouting materials for shield tunneling. Dev. Built Environ..

[11] Zhang C., Fu J.Y., Yang J.S., Ou X., Ye X., Zhang Y. (2018). Formulation and performance of grouting materials for underwater shield tunnel construction in karst ground. Constr. Build. Mater..

[12] Kim M., Corapcioglu M.Y. (2022). Gel barrier formation in unsaturated porous media. J. Contam. Hydrol..

[13] Mohammed A.A., Ahmed H.U., Mosavi A. (2021). Survey of mechanical properties of geopolymer concrete: A comprehensive review and data analysis. Materials.

[14] Li J., Dang X.T., Zhang J.W., Yi P., Li Y. (2023). Mechanical Properties of Fly Ash-Slag Based Geopolymer for Repair of Road Subgrade Diseases. Polymers.

[15] Sun Z.H., Voigt T., Shah S.P. (2006). Rheometric and ultrasonic investigations of viscoelastic properties of fresh Portland cement pastes. Cem. Concr. Res..

[16] Li L., Sun H.-X., Zhang Y., Yu B. (2021). Surface cracking and fractal characteristics of bending fractured polypropylene fiber-reinforced geopolymer mortar. Fractal Fract..

[17] Canakci H., Güllü H., Dwle M.I.K. (2018). Effect of glass powder added grout for deep mixing of marginal sand with clay. Arab. J. Sci. Eng..

[18] Lin C., Wang M., Liu X., Li Z., Zhang J., Gao Y. (2023). Working performance of red mud-based grouting materials mixed with ultrafine limestone and quartz. Constr. Build. Mater..

[19] Taher S.M.S., Saadullah S.T., Haido J.H., Tayeh B.A. (2021). Behavior of geopolymer concrete deep beams containing waste aggregate of glass and limestone as a partial replacement of natural sand. Case Stud. Constr. Mater..

[20] Güllü H., Cevik A., Al-Ezzi K.M.A., Gülsan M.E. (2019). On the rheology of using geopolymer for grouting: A comparative study with cement-based grout included fly ash and cold bonded fly ash. Constr. Build. Mater..

[21] Wang Y., Liu X., Tang B., Li Y., Zhang W., Xue Y. (2021). Effect of Ca/(Si + Al) on red mud based eco-friendly revetment block: Microstructure, durability and environmental performance. Constr. Build. Mater..

[22] Ortega J.M., Cabeza M., Tenza-Abril A.J., Real-Herraiz T., Climent M.Á., Sánchez I. (2019). Effects of red mud addition in the microstructure, durability and mechanical performance of cement mortars. Appl. Sci..

[23] Karlina A.I., Karlina Y.I., Gladkikh V.A. (2023). Analysis of experience in the use of micro- and nanoadditives from silicon production waste in concrete technologies. Minerals.

[24] Zhao X., Liu C., Zuo L., Wang L., Zhu Q., Wang M. (2019). Investigation into the effect of calcium on the existence form of geopolymerized gel product of fly ash based geopolymers. Cem. Concr. Compos..

[25] Li Z.F., You H., Gao Y.F., Wang C., Zhang J. (2021). Effect of ultrafine red mud on the workability and microstructure of blast furnace slag-red mud based geopolymeric grouts. Powder Technol..

[26] Yang Q., Geng P., Wang J.X., Chen P., He C. (2021). Research of asphalt-cement materials used for shield tunnel backfill grouting and effect on anti-seismic performance of tunnels. Constr. Build. Mater..

[27] Li S.C., Zhang J., Li Z.F., Liu C., Chen J. (2021). Feasibility study on grouting material prepared from red mud and metallurgical wastewater based on synergistic theory. J. Hazard. Mater..

[28] Kaya-Özkiper K., Uzun A., Soyer-Uzun S. (2021). Red mud- and metakaolin-based-geopolymers for adsorption and photocatalytic degradation of methylene blue: Towards self-cleaning construction materials. J. Clean. Prod..

[29] Payakaniti P., Chuewangkam N., Yensano R., Pinitsoontorn S., Chindaprasirt P. (2020). Changes in compressive strength, microstructure and magnetic properties of a high-calcium fly ash geopolymer subjected to high temperatures. Constr. Build. Mater..

[30] Li Z.F., Liu X.L., Gao Y.F., Zhang J. (2023). Study on the hardening mechanism of Bayer red mud-based geopolymer engineered cementitious composites. Constr. Build. Mater..

[31] Xu G., Tian Q., Miao J., Liu J. (2017). Early-age hydration and mechanical properties of high-volume slag and fly ash concrete at different curing temperatures. Constr. Build. Mater..

[32] Zhang P., Kang L., Zheng Y., Zhang T., Zhang B. (2022). Influence of SiO_2_ /Na_2_O molar ratio on mechanical properties and durability of metakaolin-fly ash blend alkali-activated sustainable mortar incorporating manufactured sand. J. Mater. Res. Technol..

